# Du Diagnostique Anatomique des Maladies du Foie et de sa valeur au point de vue thérapeutique. Thèse de Concours

**Published:** 1845-07

**Authors:** 


### Art. XX.

*Du Diagnostique Anatomique des Maladies du Foie, et de sa valeur au point de vue thérapeutique. Thèse de Concours.* Par Maxime Vernois, m.d.—*Paris,* 1844. 8vo, pp. 84.

The phrase anatomical diagnosis being comparatively new, M. Vernois considers it a matter of necessity to define the words, and does so by stating them to signify that species of diagnosis which is applied to the "local determination of the seat, extent, and nature of the diseases of any organ, and which in order to attain its end, can only have recourse to physical characters appreciable by the senses." Here we have obviously the "physical diagnosis" of various authors in our own country; and the British term seems to us, of the two, unquestionably the more precise and significant of the thing meant.

It is to be remembered the question which the chances of the *concours* gave the author to handle is to what extent this kind of diagnosis applied to the liver and gall-bladder, is capable of lending guidance in the *treatment* of diseases of those organs.

After a few introductory flourishes, M. Vernois attacks his subject by giving an outline of the physical means whereby the natural condition of the liver and gall-bladder may be established. He "purposely omits *inspection* of the region of the liver; believing that in the state of health, this method of examination furnishes no help in determining the form and dimensions of the organ. Here is an exposure of complete ignorance of the observations of Dr. Edwin Harrison, observations proving the precise reverse of the position thus heedlessly laid down. But M. Vernois is, doubtless, too good a disciple of the Paris school, to fancy for an instant that anything worth being known could emanate from "nous autres pauvres insulaires."

Percussion is the true method of ascertaining the outline of the liver, and "to M. Piorry are due *all* existing researches on the subject." Now we have certainly borne our testimony more than once in this Journal, to the merits of the indefatigable percussor, and cannot be therefore supposed to be quite ignorant of their nature and scope, but we do confess this piece of outrageous absurdity startled us, and appeared inexplicable until we discovered that M. Piorry was one of the judges of the concours,—immaculate, unbending judges, upon whose ears flattery falls in vain. "Comme il a la tête montée, ce pauvre Piorry, depuis qu'il a été nommé Professeur," say the sneerers of the Ecole de Médecine; but in the name of frail human nature, if he often get such doses of grovelling adulation, as the specimen just given, can the poor man be expected to consider himself quite on the level of his minions?

The following passage contains the results of some inquiries recently made by M. Piorry upon percussion of the liver posteriorly:

"The limits of the liver are more difficult to determine behind than in front; the difficulty depends upon the presence of a very thick lamella of lung interposed between the liver and thoracic parietes, and besides, in respect of the inferior border, upon the presence of the kidney and of the colon filled, as this frequently is, with solid matters. The following is the manner of proceeding. Percussion should be practised in a vertical direction from above downwards, a few centimeters distance from the spine; the percussion at the upper part should be very strong, in order that the dull sound of the liver may be detected through the interposed lung. Once the upper point of dullness ascertained, percussion should be gentle and superficial. The point where the lung ceases, is soon discovered and the presence of the liver disclosed by its special dullness and resistance. The dullness of the liver is often confounded inferiorly with that of the kidney. . . . . More marked resistance of the finger, and more complete deadness of sound point to the presence of the latter organ, and separate it from the liver."

Now we have not the least difficulty in assigning to M. Rayer the merit of having much more fully, clearly, and satisfactorily pointed out the means of physically distinguishing the liver and kidney, than M. Piorry does in this passage; but M. Rayer was not a "judge," so that of course M. Vernois had nothing to do with him.

The physical methods of examination applicable to the liver and gall-bladder in a state of disease, are enumerated by M. Vernois as:—"inspection; palpation; touch; pressure of the hepatic and adjoining regions; local and comparative mensuration; percussion; auscultation; ballottement; fluctuation; particular noises, and special sensations; puncture with the exploring needle; decumbency of the patient."

The only circumstance under which, according to M. Vernois, inspection can aid in disclosing the *nature* of enlargement of the liver, is when superfical visible œdema, more or less distinctly circumscribed, coincides with that enlargement; abscess of the organ may then be suspected. In some doubtful cases this appearance might justify the observer in practising punctures or incisions,—this is the only way in which *inspection* can throw light on treatment.

The very first proposition M. Vernois lays down respecting the position of the liver as ascertainable by percussion, (although he has had the *toute puissance* of M. Piorry's plessimetric exploits to guide him,) is an error. Whenever the liver extends, even so slight a distance as three centimetres above the fifth rib, it is, he affirms, abnormally elevated. Now it has on the contrary become matter of mathematical certainty for us since we became acquainted with Dr. Edwin Harrison's method of investigating the point, that in the state of perfect health the upper boundary of the organ commonly reaches the fourth rib, or close to this.

"Bleed largely, or purge energetically a patient labouring under hyperhepatohemia, [which being interpreted means, we presume, congestion of the liver,] and in a few days or hours, *often almost immediately after*, [?] diminution in the size of the liver may *easily* be ascertained." How pretty all this looks on paper!

Auscultation, generally speaking, can afford no *direct* information concerning the condition of the liver. In the following case observed by the author in M. Andral's wards in 1834, it might (had such cases been previously observed) have helped directly to establish the diagnosis. A man aged 40, had an abdominal tumour extending from the epigastrium to the region of the liver, and furnishing under pressure a very manifest crepitating sound, such as might be supposed to arise from the displacement of fragments of solid matter. The patient died of acute peritonitis. In front of the liver an enormous cyst was discovered, containing fragments, of some size, of cretaceous matter, associated with a sort of whitish pulpy substance, the cyst had given way during life; some of its contents fell into the peritoneal sac, hence the fatal inflammation of that membrane.

With all desire to discover any further novelty in the pages of M. Vernois, we have failed to do so. This thesis, however, is not without its value, as a summary of things already known.

